# Microbiota-metabolism-epigenetics-immunity axis in cancer

**DOI:** 10.3389/fimmu.2024.1449912

**Published:** 2024-07-12

**Authors:** Bo Ren, Yuan Fang, Minzhi Gu, Lei You, Taiping Zhang, Yupei Zhao

**Affiliations:** ^1^ Department of General Surgery, Peking Union Medical College Hospital, Peking Union Medical College, Chinese Academy of Medical Sciences, Beijing, China; ^2^ Key Laboratory of Research in Pancreatic Tumor, Chinese Academy of Medical Sciences, Beijing, China; ^3^ National Science and Technology Key Infrastructure on Translational Medicine in Peking Union Medical College Hospital, Beijing, China

**Keywords:** immunotherapy, microbiota, metabolism, epigenetics, hallmarks of cancer

## Introduction

Over the past several years, the field of oncology has seen rapid advances, generating a comprehensive and intricate understanding of cancer. This body of knowledge has unveiled cancer as a disease characterized by ongoing transformations across various physiological and pathological processes. Based on these researches and findings, the hallmarks of cancer have been delineated, providing a collection of essential functional attributes that human cells undergo during their transition from a healthy state to a state of cancerous proliferation. These attributes are pivotal for the cells’ capacity to initiate and sustain the growth of malignant tumors. As of the latest updates, Prof. Hanahan and Weinberg have identified a total of 14 hallmarks that characterize cancer (1–3). These hallmarks encompass a range of cellular capabilities and adaptations, including sustaining proliferative signaling, evading growth suppressors, resisting cell death, enabling replicative immortality, and activating invasion & metastasis. They also address the tumor’s interaction with the microenvironment, such as promoting genome instability & mutation, inducing tumor-promoting inflammation, and deregulating cellular metabolism. Furthermore, they highlight the importance of avoiding immune destruction, unlocking phenotypic plasticity, undergoing nonmutational epigenetic reprogramming, and engaging with polymorphic microbiomes, as well as the role of senescent cells in cancer development.

Contrary to once being viewed as discrete, the hallmarks of cancer are now recognized as interrelated and mutually reinforcing processes. The intricate crosstalk between these hallmarks is the subject of intense investigation, as elucidating their complex interactions holds the key to understanding cancer’s adaptability and resistance to therapies. For instance, research has highlighted that the gut microbiota has the capacity to both amplify the benefits of immunotherapy by fine-tuning the body’s antitumor immune response via checkpoint inhibitors (4, 5), and also, in some cases, to impede the immune system’s ability to fight off cancer (6), underscoring the close connection between microbes and tumor immunity. Meanwhile, microbiota-derived metabolites could affect anti-tumor immunity. It was demonstrated that the maladaptation of the host-microbiota metabolic interaction, particularly the activation of the host’s urea cycle metabolism and the imbalance of beneficial and pathogenic bacteria, played a pivotal role in the development of colorectal cancer, which uncovered the interplay between microbiota, metabolism and tumor immunity. A series of studies on the role of acetyl-CoA in pancreatic cancer has revealed that KRAS mutations in pancreatic cancer mediate the production of acetyl-CoA, which subsequently upregulates the expression of oncogenes through histone acetylation, thereby promoting the development of pancreatic cancer (7, 8), emphasizing the crosstalk between nonmutational epigenetic reprogramming and cellular metabolism. These insights are crucial for developing a more integrated view of cancer biology.

Recently, Jia et al. published a research article in *Cell* entitled “Microbial metabolite enhances immunotherapy efficacy by modulating T cell stemness in pan-cancer”, revealing the microbiota-metabolism-epigenetics-immunity axis in cancer. This finding promises a more holistic understanding of the crosstalk between cancer hallmarks, moving us closer to a paradigm where cancer is viewed not as a collection of independent pathologies, but as a multifaceted disease shaped by a dynamic and interconnected biological network.

## From microbiota to immunity

In order to investigate the microbiota related to sensitivity in immune checkpoint blockade (ICB) therapy, the research team established a Mc38 (colorectal cancer cell line derived from mice) subcutaneous tumor xenograft model for treatment with anti-PD-1 antibodies. Previous studies have reported that the gut microbiota could modulate immunotherapy (9, 10). The results of fecal transplantation test confirmed that the composition of gut microbiota was associated with ICB immunotherapy responsiveness. Then, they collected the fecal samples from the responder or non-responder mice and analyzed for microbial composition by 16S rRNA sequencing, revealing a significant positive association between the presence of Lactobacillus johnsonii (L.j.) and the efficacy of anti-PD-1 treatment.

## Microbiota’s metabolic impact on ICB therapy of cancer

To determine if the observed effects were due to L.j. itself or its metabolic byproducts, the researchers conducted a series of experiments. They treated mice with various forms of L.j., including heat-killed bacteria, sonically disrupted samples, the original growth medium (MRS), and the conditional culture medium (Lj. CM), alongside live cultures of L.j. Notably, it was the group treated with the conditional medium (Lj. CM + anti-PD-1) that exhibited an immunotherapeutic response similar to that of the group treated with live L.j. (L. j + anti-PD-1), suggesting that the metabolites derived from L.j. contributed to bolster the response to ICB therapy. By utilizing plasma liquid chromatography-tandem mass spectrometry (LC-MS/MS), they identified significant enhancement in tryptophan metabolism in the group treated with live L.j. The experiments utilizing a tryptophan-deficient diet have confirmed the indispensable role of tryptophan in the L.j.-mediated promotion of ICB therapy responsiveness. Subsequently, the research team employed targeted metabolomics to identify indole-3-propionic acid (IPA) as a key metabolite in the tryptophan metabolism pathway. This was followed by a series of *in vivo* experiments that affirmed IPA’s capacity to augment the efficacy of immune checkpoint blockade (ICB) therapy. Notably, treatment with IPA led to an increased infiltration of CD8^+^ T cells into the tumor microenvironment, thereby enhancing the responsiveness to ICB treatment, which was not observed in Rag1-deficient mice (the mice lack mature B and T cells). Together, the above findings indicated that L.j.-derived IPA could promote the responsiveness to ICB therapy, dependent on CD8^+^ T cells.

However, tryptophan is typically metabolized through the pathway involving indole-3-pyruvate acid (IPYA), indole-3-lactic acid (ILA), indole-3-acrylic acid (IA), and ultimately IPA (11). In the conditional medium of L. johnsonii, only ILA was detected, and experiments showed that ILA alone could not sensitize ICB therapy. Consequently, the research team delved further into the reasons behind L. johnsonii’s production of IPA, discovering that C. sporogenes (C. s.) could convert ILA into IPA. This finding was corroborated through corresponding animal experiments, which confirmed that the production of IPA by L. johnsonii is contingent upon the metabolic activity of C. sporogenes.

## The epigenetic bridge between metabolism and immunity

To elucidate the specific mechanisms by which IPA sensitizes ICB therapy through CD8^+^ T cells, the research team conducted single-cell RNA sequencing (scRNA-seq), single-cell T cell receptor sequencing (scTCR-seq), and single-cell ATAC sequencing (scATAC-seq) analyses on CD8^+^ T cells from tumor-bearing mice. The findings revealed that IPA reduces the proportion of naive CD8^+^ T cells while increasing the ratios of progenitor exhausted T cells (T_pex_) and effector T cells (T_eff_). Conditional knockout mouse experiments with TCF7—a marker for T_pex_ cells—demonstrated that the sensitization of ICB therapy by IPA is dependent on T_pex_ cells. Given previous reports that T_pex_ cells are primarily regulated by histone modifications (12), the authors performed an integrated analysis of scRNA-seq and scATAC-seq data, uncovering that IPA upregulates the chromatin accessibility at the super-enhancer of the Tcf7 gene. Subsequently, further confirmation was achieved through Chromatin Immunoprecipitation (ChIP), Cleavage Under Targets and Release Using Nuclease (CUT&RUN), and Cleavage Under Targets and Tagmentation (CUT&Tag) assays, which substantiated that IPA enhances the level of H3K27 acetylation at the Tcf7 super-enhancer. In summary, IPA, produced by L. johnsonii and C. sporogenes, upregulates Tcf7 expression through histone acetylation, promotes the differentiation of CD8^+^ T cells into T_pex_, and thereby strengthens anti-tumor immunity and the responsiveness to ICB therapy. These results collectively confirm the existence of the microbiota-metabolism-epigenetics-immunity axis, highlighting its critical role in the modulation of cancer immunotherapy.

## Microbiota-derived IPA in immunotherapy of CRC organoids and other types of cancer

In the culmination of their study, the authors explored the role of microbiota-derived IPA in enhancing the efficacy of ICB therapy at a pan-cancer level. Utilizing transplantable models of breast cancer and melanoma, as well as the MMTV-PyMT spontaneous breast cancer model and the cecum orthotopic implantation model, they further confirmed that IPA can increase the infiltration of T_pex_ cells within the tumor microenvironment, thereby sensitizing ICB treatment for breast, melanoma, and colorectal cancers. Additionally, the research team established an air-liquid interface (ALI) patient-derived organoids (PDOs) system, which includes a more comprehensive immune microenvironment and additional matrix components, allowing for a more precise representation of immunotherapy dynamics (13). Within the ALI-PDOs, it was similarly observed that IPA could enhance the infiltration of CD8^+^ T cells in tumors and upregulate the expression of effector proteins in T_eff_ cells. These findings collectively validate the potential of microbiota-derived IPA to reinforce the effectiveness of tumor ICB therapy across various cancer types, laying a robust foundation for its potential clinical application.

## Discussion

**Figure 1:**
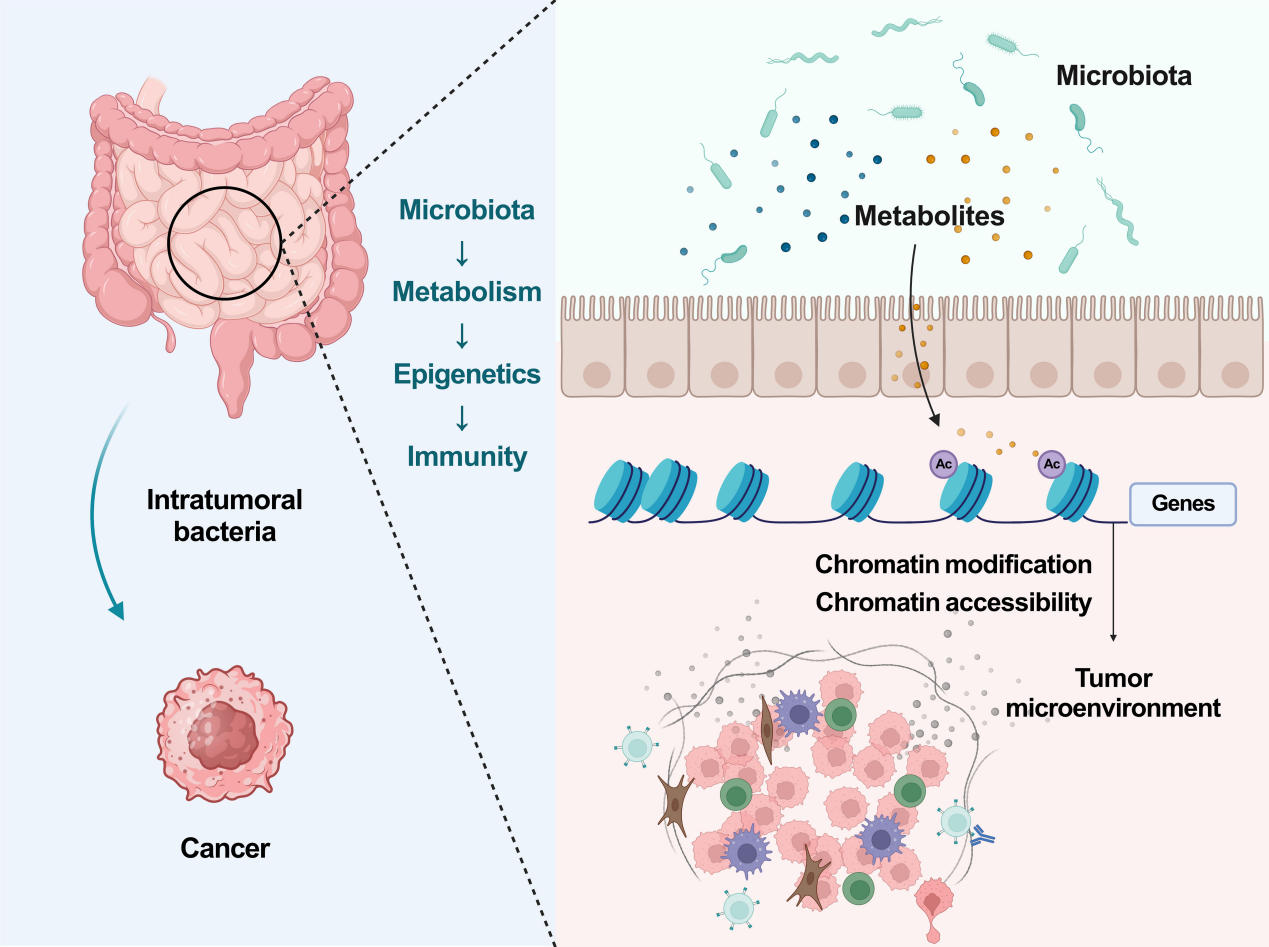
The microbiota-metabolism-epigenetics-immunity axis in cancer. Ac, Histone acetylation.

This study presents a groundbreaking revelation that the gut microbiota, specifically L.j. and C.s., can synthesize IPA to enhance the infiltration of CD8^+^ T cells into the tumor microenvironment. The augmentation of T_pex_ and T_eff_ cell populations through histone acetylation mechanisms significantly sensitizes cancer to ICB therapy. The findings are robust and solid. Most importantly, this study also provide substantial evidence supporting the role of the microbiota-metabolism-epigenetics-immunity axis in modulating cancer immunotherapy responses (**Figure 1**).

Previous studies have laid the groundwork for understanding the microbiota’s role in cancer. Early research established the gut microbiota’s influence on cancer development, with subsequent studies revealing its impact on the efficacy of chemotherapy and radiotherapy. More recent investigations have highlighted the microbiota’s capacity to modulate the immune response, particularly in the context of ICB therapy. The discovery that specific microbial metabolites, such as butyrate (14) and indole-3-lactic acid (ILA) (15), can directly shape the epigenetic landscape of immune cells, particularly CD8^+^ T cells, has opened new horizons in our understanding. These metabolites not only enhance the cytotoxic T cell response but also reprogram tumor metabolism, potentially reversing therapeutic resistance. The identification of Lactobacillus iners (16) and its role in conferring chemoradiation resistance through lactate-induced metabolic rewiring, as well as the ameliorative effects of L. plantarum-derived ILA on tumorigenesis, underscores the microbiota’s metabolic byproducts as key regulators of the tumor microenvironment. This study synthesizes these insights, offering a comprehensive perspective on how the microbiota’s metabolic output can be harnessed to fine-tune immunotherapies and improve patient outcomes.

The findings from this study point towards several promising directions for future research. First, in addition to the gut microbiota, the recently discovered intratumoral microbiota (17) merits attention and exploration for its role in the progression and therapy of cancer. Second, there is a need to further explore the specific mechanisms by which microbiota-derived metabolites interact with the host’s metabolic and epigenetic machinery. Third, the development of strategies to modulate the gut microbiota for therapeutic benefit, such as through probiotics or dietary interventions, warrants investigation. Finally, the translational potential of these findings into clinical practice, including the use of IPA as an adjuvant in ICB therapy, must be rigorously evaluated in clinical trials.

In conclusion, the research by Jia et al. provides a compelling case for the microbiota-metabolism-epigenetics-immunity axis in cancer. The study’s findings not only enhance our understanding of the intricate relationship between the microbiota and cancer immunotherapy but also offer a foundation for the development of new therapeutic strategies. As we continue to unravel the complexities of this axis, we move closer to a paradigm where cancer is viewed as a multifaceted disease shaped by a dynamic and interconnected biological network, offering a more holistic approach to cancer treatment.

## Author contributions

BR: Writing – original draft. YF: Writing – original draft. MG: Writing – original draft. LY: Writing – review & editing. TZ: Writing – review & editing. YZ: Writing – review & editing.
